# Expression of the extracellular sulfatase SULF2 is associated with squamous cell carcinoma of the head and neck

**DOI:** 10.18632/oncotarget.9506

**Published:** 2016-05-20

**Authors:** Sarah A. Flowers, Xin Zhou, Jing Wu, Yiwen Wang, Kepher Makambi, Bhaskar V. Kallakury, Mark S. Singer, Steven D. Rosen, Bruce Davidson, Radoslav Goldman

**Affiliations:** ^1^ Department of Oncology, Lombardi Comprehensive Cancer Center, Georgetown University, Washington, DC 20057, USA; ^2^ Department of Pathology, Lombardi Comprehensive Cancer Center, Georgetown University, Washington, DC 20057, USA; ^3^ Department of Anatomy, University of California, San Francisco, CA 94143, USA; ^4^ Department of Otolaryngology-Head and Neck Surgery, Medstar Georgetown University Hospital, Washington, DC 20057, USA; ^5^ Department of Biochemistry and Molecular and Cellular Biology, Georgetown University, Washington, DC 20057, USA

**Keywords:** heparan sulfate proteoglycans, biomarker, sulfation, cancer progression, tumorigenesis

## Abstract

Sulfatase 2 (SULF2), an extracellular sulfatase that alters sulfation on heparan sulfate proteoglycans, is involved in the tumorigenesis and progression of several carcinomas. SULF2 expression has not been evaluated in squamous cell carcinoma of the head and neck (HNSCC). Here we report results of IHC of SULF2 expression in HNSCC tissue. SULF2 was detected in 57% of tumors (*n* = 40) with a significant increase in intensity and number of stained cells compared to adjacent cancer-free tissue (*p*-value < 0.01), increasing with cancer stage when comparing stages 1 and 2 to stages 3 and 4 (*p*-value 0.01). SULF2 was not detected in epithelial cells of cancer-free controls, and expression was independent of patient demographics, tumor location and etiological factors, smoking and HPV infection by p16 IHC analysis. Sandwich ELISA was performed on serum of HNSCC patients (*n* = 28) and controls (*n* = 35), and although SULF2 was detectable, no change was observed in HNSCC. Saliva, collected by mouthwash, from HNSCC patients (*n* = 8) and controls (*n* = 8) was also tested by ELISA in a preliminary investigation and an increase in SULF2 was observed in HNSCC (*p*-value 0.041). Overall, this study shows that SULF2 is increased in HNSCC independent of tissue location (oral cavity, oropharynx, larynx and hypopharynx), patient demographics and etiology. Although no change in SULF2 was detected in HNSCC serum, its detection in saliva makes it worthy of further investigation as a potential HNSCC biomarker.

## INTRODUCTION

HNSCC is the sixth leading cause of cancer death worldwide with over 500 000 cases annually [1] and a 5 year survival rate of 50% [2]. Early detection of HNSCC is a key factor for improving the survival rate of HNSCC patients; however, a high proportion of patients are diagnosed at an advanced stage [3]. The heterogeneous nature of HNSCC location and smoking or HPV etiology further strengthen the need for improved personalized assessment of the disease. Biomarkers from an accessible sample could be valuable in screening and early detection as well as prognostic or predictive of response in identified cases. Extracellular sulfatases, Sulfatase 2 (SULF2) and Sulfatase 1 (SULF1), have emerged as potential candidates for detection and therapeutic management of several cancers. SULF2 is of particular interest, as it is over-expressed in a range of cancers including hepatocellular [4], breast [5], pancreatic [6], non-small cell lung [7] and esophageal [8] carcinomas. Data mining of the Oncomine microarray database revealed an increase in SULF2 in brain, breast, kidney and head and neck cancers compared to healthy controls [9]. Despite this finding, SULF2 expression has not been examined in HNSCC tissue which we address in our current study.

The extracellular sulfatases (SULFs), distinct from the lysosomal sulfatases, are encoded with leader sequences and have the important extracellular function of modifying the sulfate pattern of heparan sulfate proteoglycans (HSPGs) by specifically removing the 6-*O*-sulfate from the heparan sulfate (HS) chains [9]. Therefore, together with Golgi-associated sulfotransferases involved in the biosynthesis of HS, the SULFs control the sulfation code of HSPGs which regulates the activity of many signaling pathways implicated in tumorigenesis [9]. SULF2 gene expression is developmentally and spatially regulated with the highest expression in the ovary, brain, urinary bladder and lung in mice [10]. SULF2 knock-out mice display a small reduction in litter size and body weight and a shorter life span while SULF1-deficient mice present no phenotype [11]; however, simultaneous disruption of SULF1 and SULF2 leads to perinatal lethality and developmental defects [12]. First identified for their roles in Wnt-dependent signaling during muscle development in quail [13], the SULFs have been shown to modulate pathways involving glial cell line–derived neurotrophic factor [14], vascular endothelial growth factor [15], fibroblast growth factor [16], and Noggin [17], through their actions on essential associated HSPGs.

HSPGs are glycoproteins that are located on the cell surface (glypicans, syndecans) or secreted from the cell directly into the extracellular matrix (ECM) (agrin, type XVII collagen) or as part of a secretory vesicle (serglycin) [18]. This limited set of core proteins are covalently linked with one or more heparan sulfate chains which exhibit high diversity in chain length and sulfation pattern (5 different positions) in a tissue and developmental-stage specific manner [13, 19–21]. HSPGs carry out multiple structural and signaling functions, the majority of which are associated with their ability to bind a diverse range of ligands from cytokines, growth factors, morphogens and chemokines, to proteases or their inhibitors and cell adhesion components [18]. Essential signaling functions are carried out either by acting as a co-receptor and promoting receptor signaling [18, 22–24] or by directly binding ligands [13, 25, 26].

SULF2 upregulation has been suggested to promote tumorigenesis through its action on HSPGs and SULF2 is considered to be a cancer-causing agent and a potential therapeutic target for several cancers [9]. Here, we investigated Sulf2 levels in HNSCC tissue by immunohistochemical (IHC) staining using monoclonal antibodies raised against the SULF2 protein. Further analysis was undertaken using a newly developed SULF2 sandwich ELISA [27] to determine SULF2 concentration in serum and mouthwash from HNSCC patients.

## RESULTS

### Subject characteristics

Newly diagnosed HNSCC patients (*n* = 40, Table 1) were included in this study along with healthy controls (*n* = 22) which were frequency matched on demographics of the cancer patients. The patient population was predominately male (33/40) with an average age of 54.5 ± 17.2 years. The race distribution (Caucasian 74.4%, African American 15.4% and other 10.3%) reflects the demographics seen at the Georgetown University Hospital and represents approximately the current demographics of the United States. Patients were diagnosed with HNSCC of the oral cavity (*n* = 16), oropharynx (*n* = 13), larynx (*n* = 9) or hypopharynx (*n* = 2); 12 of the patients had early stage disease (stage 1 and 2), 25 advanced disease (stage 3 and 4) and staging information was not determined for four participants.

**Table 1:** Clinicopathological and demographic characteristics of the study population

	Tissue		Serum	
	Control	HNSCC	Control	HNSCC
Number	22	40	35	28
Age (yr)	53.6 ± 9	54.5 ± 17	54.1 ± 9	54.6 ± 9
Race				
CA	18	29	25	22
AA	2	6	6	4
other	2	4	4	2
Gender				
female	5	8	10	4
male	17	32	25	24
Tumor location				
hypopharynx	NA	2	NA	1
larynx		9		6
oral cavity		16		11
oropharynx		13		10
Tumor stage				
1	NA	5	NA	5
2		7		4
3		6		3
4		19		14

CA is Caucasian and AA is African American.

### SULF2 expression in HNSCC is associated with tumor cells and increases with TNM stage

**Figure 1:**
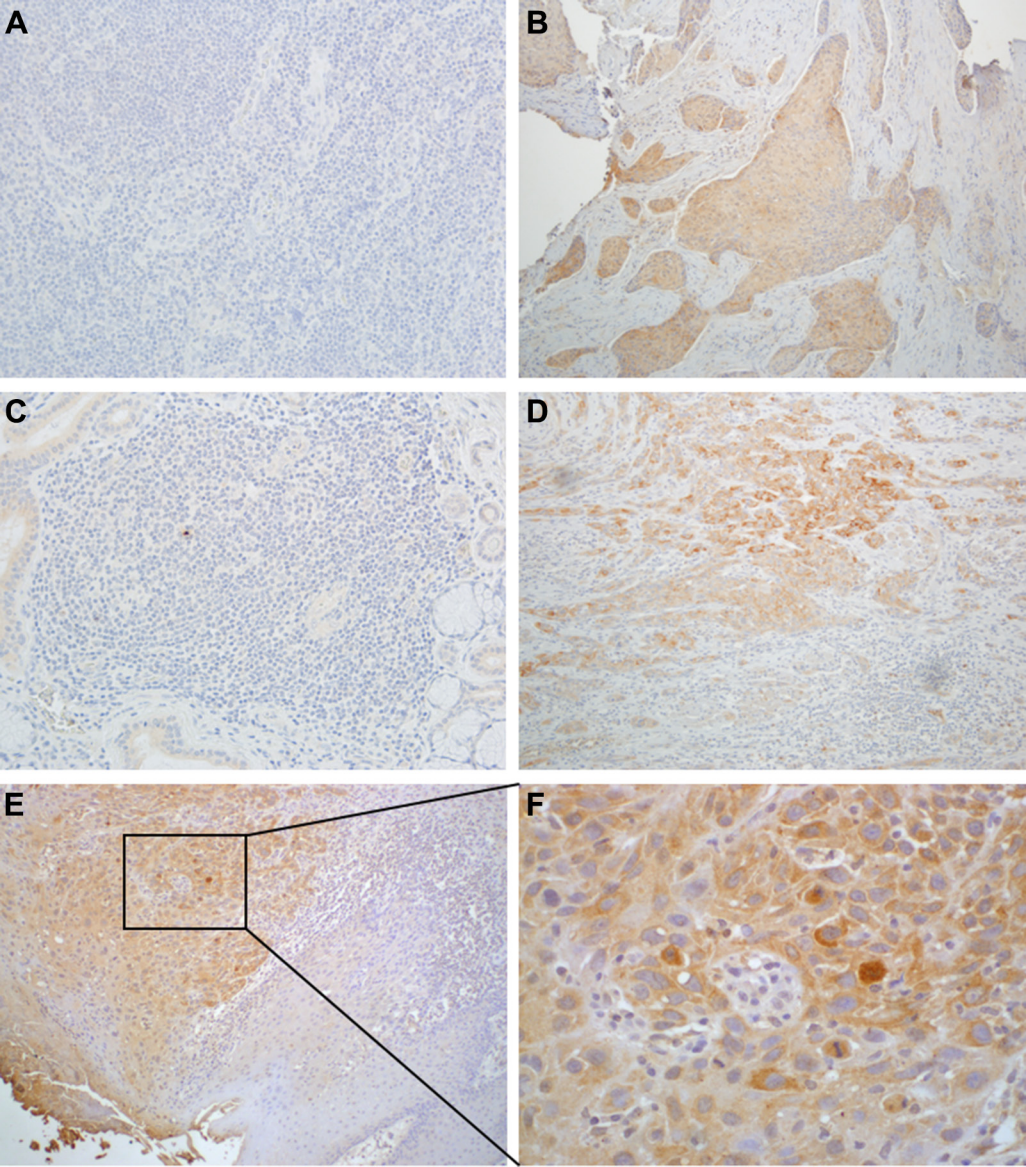
SULF2 IHC staining of HNSCC and healthy control tissues (**A**) Laryngeal tissue of a cancer-free person and (**B**) laryngeal tissue of a cancer patient. (**C**) Tissue from the oral cavity of a cancer-free person and (**D**) oral cavity of a cancer patient. In HNSCC tissue, cytoplasmic staining was significantly more intense and distributed over higher percentage of cells compared to adjacent non-cancerous fibroblasts. (**E**) SULF2 staining of tumor and adjacent normal tissue from a HNSCC patient at 10× magnification and (**F**) the same tissue at 40× magnification showing intense cytoplasmic SULF2 staining in the tumor.

HNSCC tissue sections containing both tumor and adjacent cancer-free regions were evaluated by IHC together with additional tissues of five cancer-free patients with available paraffin embedded sections. The cancer free controls were taken from the larynx, soft palate, supraglottic larynx and buccal mucosa. The five cancer free controls showed no SULF2 staining of the squamous epithelial cells (Figure 1A and 1C). SULF2 staining was, however, apparent in 23 of the 40 HNSCC tissues (Figure 1B and 1D). SULF2 staining was localized to the cytoplasm of cancer cells (Figure 1E and 1F); in addition, diffuse weak staining was observed in the extracellular stroma of the tissue of all categories and this background stain was not included in the IHC evaluation.

**Figure 2:**
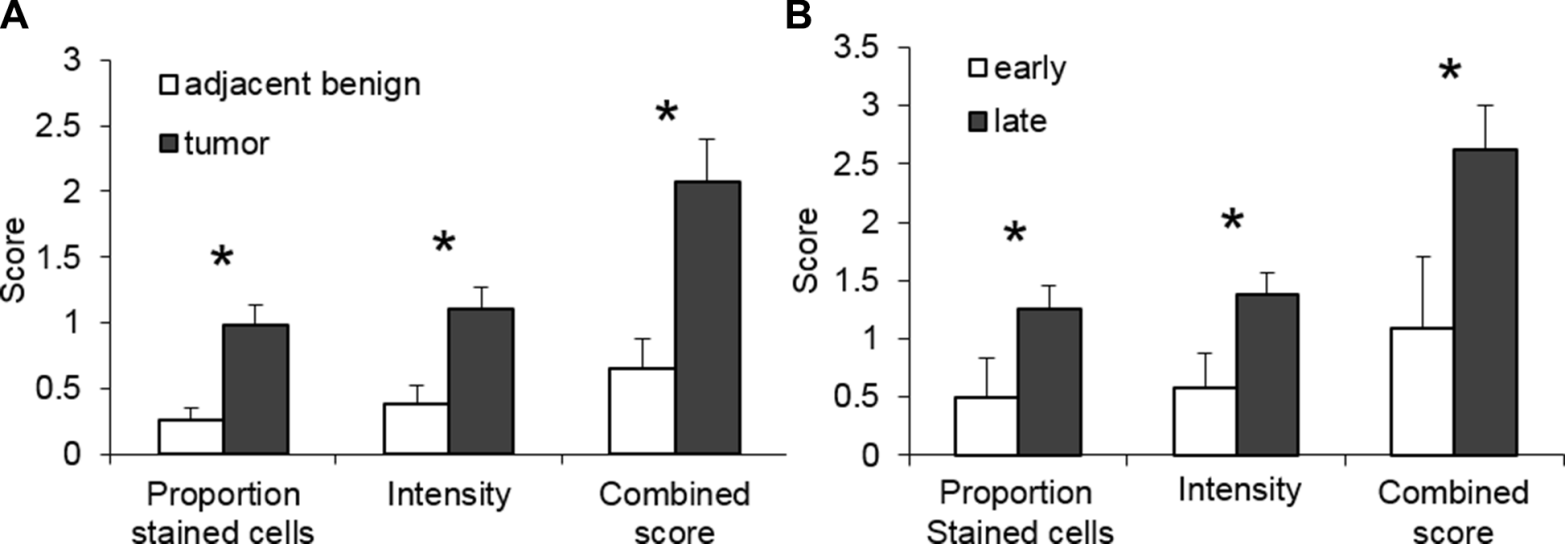
SULF2 expression is associated with cancer status (**A**) SULF2 IHC staining is increased in the cytoplasm of epithelial cells of the HNSCC tumors (*n* = 40); tumors have a higher proportion of stained cells (*p*-values = 0.0002), intensity (*p*-values = 0.002) and combined scores (*p*-values = 0.0005) than adjacent cancer-free tissue. (**B**) Late stage HNSCC tumors (stages 3 and 4, *n* = 25) have a higher proportion of stained cells (*p*-value = 0.009), intensity (*p*-value = 0.012) and combined score (*p*-value = 0.011) than early stage tumors (stages 1 and 2, *n* = 12).

To further compare SULF2 staining in HNSCC and adjacent cancer-free regions, the intensity of SULF2 staining and the proportion of stained cells were scored separately and their sum was designated as the combined score (Figure 2A). The HNSCC tumor tissues showed significantly higher SULF2 staining in all the scored categories. The proportion of cells showing SULF2 staining was significantly higher (*p*-value = 0.0002) in HNSCC tumor tissue (mean = 0.98, SD = 1.00) compared to adjacent tissue (mean = 0.26, SD = 0.51). The intensity of SULF2 staining was significantly increased (*p*-value = 0.002) in tumor cells (mean = 1.10, SD = 1.10) compared to adjacent tissue (mean = 0.40, SD = 0.76) and, consequently, the combined score was also significantly higher (*p*-value = 0.0005) in HNSCC (mean = 2.07, SD = 2.03) compared to adjacent tissue (mean = 0.65, SD = 1.25). SULF2 expression in HNSCC tissue was also associated with tumor grade (Figure 2B) with patients with advanced disease (stages 3 and 4) expressing a significantly higher intensity of SULF2 staining (1.38 vs 0.58, *p* = 0.043) and an increased proportion of stained cells (1.25 vs 0.50, *p* = 0.042) compared to patients with early stage HNSCC (stage 1 and 2).

### SULF2 expression is not affected by tumor location, patient demographic characteristics, smoking or HPV infection

**Figure 3:**
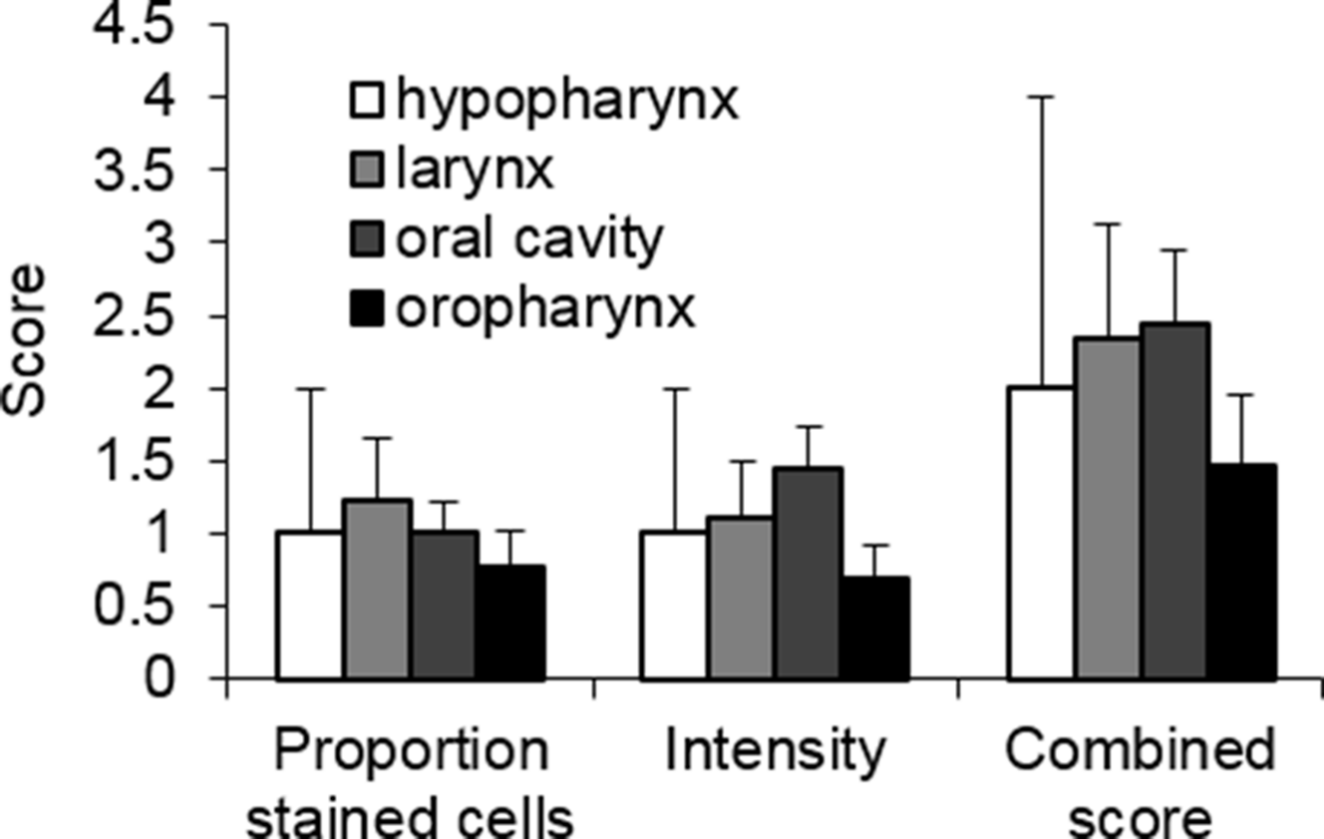
SULF2 expression in HNSCC from different locations measured by IHC staining The average SULF2 IHC scores of HNSCC tumors from four different locations, hypopharynx (*n* = 2), larynx (*n* = 9), oral cavity (*n* = 16) and oropharynx (*n* = 13), were compared. IHC staining was scored based on the proportion of cells stained and intensity with the sum of these two scores designated the combined score. Error bars represent standard error of the mean.

We ascertained if SULF2 expression was affected by tumor properties or characteristics of the patients. SULF2 staining was compared between the four HNSCC sites analyzed including the hypopharynx (*n* = 2), larynx (*n* = 9), oral cavity (*n* = 16) and oropharynx (*n* = 13). The tumor location had no statistically significant (*p* value > 0.05) effect on the intensity of SULF2 expression or the proportion of cells that showed staining in the four sites tested (Figure 3). However, the number of tissues are small and some variation was observed with oropharyngeal tissues showing the lowest SULF2 expression using all three scores and the oral cavity showing the highest intensity and combined scores. Patient demographics were also considered including age, race and gender (Supplementary Figure S1), however, no statistically significant association with SULF2 IHC staining was identified for any of these characteristics.

Smoking has historically been the major HNSCC risk factor, but HPV infection has emerged as strongly associated with a subset of the HNSCC tumors, most notably those at oropharyngeal sites. The expression of p16 is associated with HPV infection and used as a marker of HPV infection [28]. Of the HNSCC cases with enough tissue to allow testing for HPV by IHC analysis (*n* = 35), 24 cases were negative and 11 were identified as p16 positive, of which 10 were from the oropharynx and one from the larynx (Figure 5A and 5B). A comparison of SULF2 expression in p16 positive and p16 negative HNSCC cases (Figure 5C) showed no statistically significant difference in SULF2 intensity (*p*-value = 0.23) or the proportion of cells stained with the p16 antibody (*p*-value = 0.83). The SULF2 IHC staining pattern also showed no statistically significant difference in tumor tissue or adjacent cancer-free tissue between non-smokers and smokers when categorized as patients who were currently smoking, as well as patients who had ever been smokers (Supplementary Figure S2).

**Figure 4:**
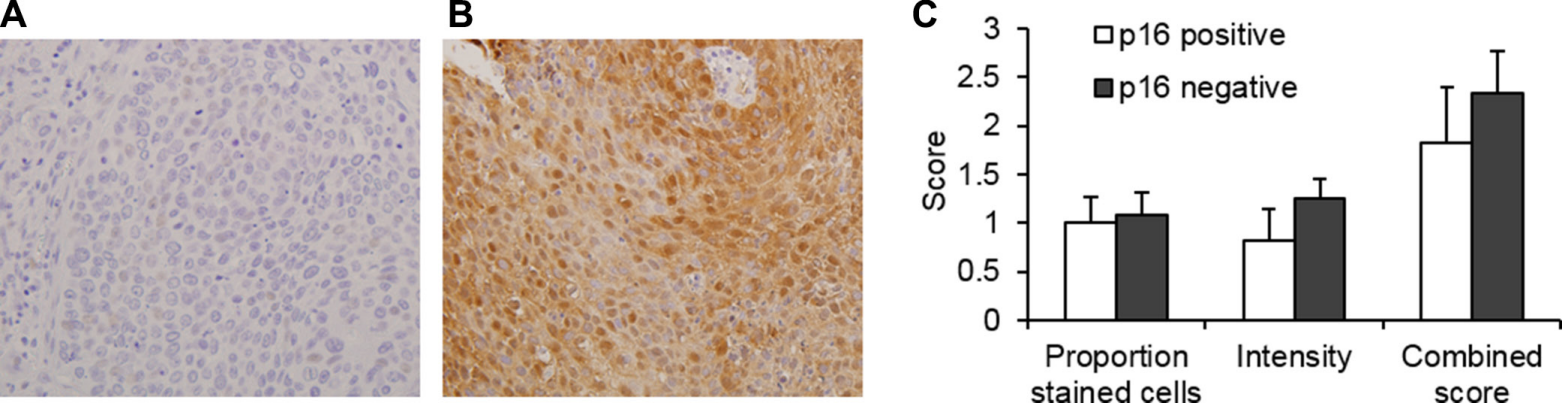
SULF2 expression is not associated with p16 status in HNSCC An example of IHC staining of (**A**) p16 negative and (**B**) p16 positive oropharyngeal tissue. (**C**) The average p16 IHC scores of p16 positive (*n* = 11) and p16 negative (*n* = 24) HNSCC tumors were compared. IHC staining was scored based on the proportion of cells stained and intensity with the sum of these two scores designated the combined score. Error bars represent standard error of the mean.

**Figure 5:**
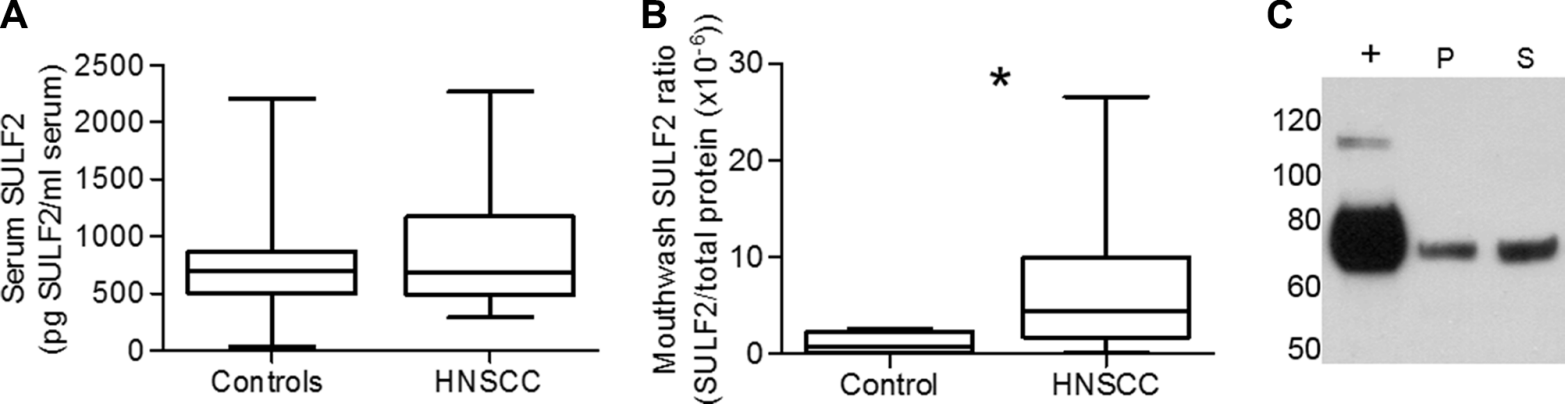
(**A**) Comparison of serum SULF2 concentration quantified by ELISA in HNSCC patients (*n* = 28) and cancer-free controls (*n* = 35). (**B**) SULF2 was quantified by ELISA from mouthwash samples. Head and neck cancer patients (*n* = 8) have higher SULF2 contents in mouthwash proteins than healthy controls (*n* = 8, *p*-value = 0.041). Graph whiskers represent the minimum and maximum, box extends from the 25th to 75th percentile and line represents the median. (**C**) Western blot detection of SULF2 (mAb 8G1) in mouthwash from HNSCC patient mouthwash pellet (P) and mouthwash Supernatant (S). Positive control of SULF2 expressing MCF7 cell media (+) also shown.

### SULF2 is not elevated in serum of HNSCC patients but is detectable in mouthwash

Given the biomarker potential of SULF2, we used our newly established ELISA assay to evaluate SULF2 in serum (Figure 5A). SULF2 was present in low but detectable levels in sera of both HNSCC patients and healthy controls. Although the HNSCC serum samples showed a higher mean concentration of SULF2 (*n* = 28, mean = 849 pg/ml serum, SD = 491) compared to control samples (*n* = 35, mean = 732 pg/ml serum, SD = 412), we did not observe a significant difference (*p*-value = 0.56) in the SULF2 concentration. Serum concentration of SULF2, however, increased with age (*r* = 0.47, *p*-value < 0.001, Supplementary Figure S3), when HNSCC and control samples were combined, as has been previously shown [27]. No significant difference was observed with respect to race or gender, although, plasma concentration of SULF2 in women (*n* = 14, mean = 650, SD = 326) tended to be lower than in men (*n* = 48, mean = 825, SD = 475).

Due to the proximity to the tumor location, SULF2 was then evaluated in mouthwash samples available for a subset of patients. These samples were collected by a 2 minute rinse of the oral cavity with Listerine (30 to 50 ml), a sampling protocol not yet optimized for protein analysis. Nevertheless, we were able to isolate sufficient protein (50–480 μg) from the soluble protein fraction isolated from 30 to 50 ml of mouthwash for quantification of SULF2 by ELISA. The amount of SULF2 detected was normalized to the total protein determined by Bradford assay. The normalized SULF2 values for HNSCC patients (*n* = 8) were compared to cancer free controls (*n* = 8) and tended towards (*p*-value = 0.041) a higher ratio (mean = 7.41 × 10^−6^, SD = 8.59 × 10^−6^) compared to control samples (1.20 × 10^−6^, SD = 1.09 × 10^−6^) (Figure 5B), with some HNSCC patients having up to 10 x more SULF2 than the control mean. The presence of SULF2 in these samples was confirmed by western blot compared to a positive control of SULF2 from MCF7 cell media (Figure 5C). In addition to the supernatant, SULF2 could also be detected in the mouthwash pellet fraction in some patient samples as the 75 kDa amino-terminal SULF2 fragment recognized by the 8G1 mAb under reducing conditions (Figure 5C). Although we were able to confirm the presence of SULF2 in the mouthwash of some HNSCC patients, this initial analysis does not include enough patient samples collected under optimized conditions to be considered a definitive study of its biomarker potential.

## DISCUSSION

The IHC analysis undertaken in this study showed a statistically significant increase in the expression of SULF2 in HNSCC tumor tissues compared to adjacent cancer-free tissue. SULF2 was detected in the cytoplasm of epithelial cells in 58% of patients with HNSCC by IHC analysis. Adjacent non-tumor segments showed no or weak cytoplasmic SULF2 staining and staining was not detected in the epithelial cells of cancer-free controls (*n* = 5) from the larynx, supraglottic larynx, soft palate or buccal mucosa. The 8G1 antibody used recognizes an epitope on the amino-terminal fragment of SULF2 and therefore recognizes and cannot distinguish between the domain on its own as well as the processed and unprocessed SULF2 protein. Comparison of four anatomical locations (hypopharynx, larynx, oropharynx, and oral cavity) showed no significant difference; however, the proportion of stained cells and intensity of cytoplasmic staining was lowest in the hypopharyngeal region and highest in the oral cavity while the proportion of SULF2 stained cells was slightly higher in the larynx, though, overall differences were small. Patient demographics including age, gender and race were also analyzed and no relationship between any of these parameters and SULF2 intensity or distribution was apparent, however, a larger study size may be necessary to identify subtle differences. Alteration in SULF2 expression due to major HNSCC risk factors including tobacco smoking [29] and HPV infection [30] were also considered. IHC analysis of p16 expression is currently used as a marker of HPV infection with increasing evidence that it has prognostic value. In oropharyngeal cancers, tumors of HPV etiology show better survival [31, 32] although persistent HPV detection after treatment has been associated with poor prognosis [33]. Our results indicate no association between tumor SULF2 expression levels and p16 staining (Figure 4). Smoking, evaluated as ever or current smoking, showed no correlation with SULF2 expression in tumor or tumor adjacent tissues. This indicates that smoking does not correlate with cytoplasmic SULF2 in cancer tissue or tissues adjacent to tumors; however, we did not evaluate whether smoking is correlated with SULF2 concentrations in the matrix of normal or tumor tissues because IHC staining in the stromal regions is difficult to quantify.

Upregulation of SULF2, as shown here in HNSCC, has been reported in a range of tumors including pancreatic adenocarcinoma [5, 6], hepatocellular carcinomas [4], lung adenocarcinoma, lung squamous cell carcinoma [4, 7], esophageal carcinomas [8] and human breast cancer and hyperplastic tissues [5]. Of particular interest are the squamous lung and esophageal cancers, which derive from a similar cell type and from regions anatomically related to HNSCC. Cytoplasmic staining has been observed previously in the case of lung cancer [7].

Our results show a significant association between increased SULF2 distribution and intensity in tumor tissue and cancer stage; SULF2 increases from early (stage 1 and 2) to advanced (stage 3 and 4) tumors approximately 2-fold, suggesting that SULF2 may be involved in cancer progression. Although our dataset is too small to carry out survival analysis, an association between increased SULF2 and poor outcome has been shown in other studies including a 13% increase in hazard ratio in esophageal cancer [8] and in multiple myeloma where SULF2 gene expression was shown to be an indicator of progression and poor prognosis [34]. High SULF2 expression in hepatocellular carcinoma tumor tissue has also been shown to worsen prognosis and increase recurrence after surgery [4]. SULF2 overexpression in prostate cancer cell lines has also been shown to increase cell migration and the expression of markers of epithelial to mesenchymal transition, changes important for metastasis [35].

The involvement of SULF2 in tumorigenesis has been suggested through its action on the Wnt signaling pathway which can promote tumorigenesis when dysregulated [36]. The mechanism proposed suggests that an increase in SULF2, and subsequent removal of 6-*O*-sulfate groups from HSPGs by the enzyme, reduces Wnt ligand binding sites leaving an abundance of free Wnt ligand [37]. The resulting increased pathway activation by Wnt ligand proteins leads to the accumulation of β-catenin and ultimately the increased expression of transcription factors and cell-cycle control proteins involved in tumorigenesis including Cyclin D [36, 37]. Cyclin D has been shown to be upregulated in HNSCC, particularly those not associated with HPV [3, 38]. Interestingly, the epidermal growth factor receptor (EGFR), an important pathway in HNSCC and current target for treatment [39], and Wnt signaling pathways have potential crosstalk via β-catenin [40]. Perhaps this interplay may be involved in the currently unexplained primary or acquired resistance observed with EGFR antagonist treatment using cetuximab [39]. Further investigation is essential to understand the interplay of these two important pathways in HNSCC.

Given the clear increase of SULF2 in tumor tissue and its secreted nature, SULF2 in the serum of HNSCC patients was determined. A sandwich ELISA method, using a pair of SULF2 monoclonal antibodies, previously shown to be able to detect SULF2 in normal serum was used [27]. The population analyzed in this study showed no significant difference in serum SULF2 between the healthy controls and HNSCC patients. Nonetheless, an increase in SULF2 with age was apparent, as has previously been observed in a healthy control population [27]. Other demographic parameters did not alter SULF2 serum levels; however, female HNSCC patients showed a trend towards a lower serum SULF2 compared to males. The small number of women in our study of limited size prevents definitive conclusions.

A pilot study was then undertaken to determine if SULF2 could be detected in saliva. The novel approach of using mouthwash was undertaken as the ease and noninvasive nature of collecting mouthwash makes it an attractive, underutilized sample for research and clinical applications [41, 42]. Tumor and HPV DNA have been shown to be detectable in salivary rinses from HNSCC patients with tumors of the oral cavity (100% detection) as well as from the larynx (70%), hypopharynx (67%) and oropharynx (47%) [43]. Here we demonstrate that SULF2 is detectable in the mouthwash pellet and supernatant by Western blot. Consistently quantifiable amounts of SULF2 were isolated from mouthwash supernatant for ELISA and our initial analysis shows a trend (*p*-value = 0.041) towards an increase in salivary SULF2 in HNSCC (*n* = 8) compared to controls (*n* = 8). This preliminary analysis shows that SULF2 is present in saliva of some cancer patients but we cannot perform, at this time, a definitive assessment of SULF2 in mouthwash due to a lack of sufficient patient samples collected under standard optimized sampling conditions. Further studies are needed to evaluate whether quantification of salivary SULF2 could be used for assessment of disease risk, prediction, or prognosis.

Overall, we have shown that SULF2 is over-expressed in HNSCC tumor tissue and increases in intensity and distribution in late stage tumors. SULF2 expression in the tissue was unaffected by patient demographics, tumor location or etiological factors including smoking and HPV infection. While this upregulation of SULF2 was not associated with an increase in serum concentration, a preliminary study was undertaken and SULF2 was detectable in the mouthwash of a subset of patients using the ELISA method as well as by Western blot. These observations suggest that saliva may be an effective SULF2 sampling approach worthy of further analysis.

## MATERIALS AND METHODS

### Study subjects and samples

All participants were enrolled with Informed consent obtained between 2003 and 2010 in collaboration with clinicians at the Department of Otolaryngology-Head and Neck Surgery at Georgetown University Hospital under protocols approved by the Georgetown University Institutional Review Board. Patients with newly diagnosed HNSCC undergoing surgical resection (*n* = 40) were recruited into the study. HNSCC diagnosis was made by the attending physician based on complete medical examination and was confirmed by histopathological evaluation of the HNSCC tissue. Cancer was classified according to the 7th Edition of the American Joint Committee on Cancer Staging manual. Cancer-free controls were apparently healthy visitors to Georgetown University Hospital accompanying patients coming for treatment or routine checkups. Cancer-free controls were frequency matched to the HNSCC patients on age, gender, race and smoking status defined as current, ex, or never smoking categories. All participants donated a tube of blood and some patients donated a mouthwash sample prior to surgical, radiation, or chemotherapy treatments. Serum was collected in BD Vacutainer Serum Blood Collection Tubes and isolated within 6 hours of blood collection, aliquoted, and stored at −80°C until evaluation. All assays were performed on second thaw. A subset of patients from the control serum group used here have been used in a previous study [27]. Mouthwash samples were collected, following a brief rinse with water, in Listerine which the patients held in their mouth for approximately 2 minutes. Basic characteristics of the study participants are summarized in Table 1. Sample size was not determined statistically prior to experimentation.

### Immunohistochemical staining and scoring

Paraffin embedded tissue blocks were cut into serial 5 μm sections. The first section was stained with hematoxylin and eosin and examined by the study pathologist for tissue quality and tumor content. Formalin fixed sections were de-paraffinized with xylenes and rehydrated through a graded alcohol series. Heat induced epitope retrieval was performed by immersing the tissue sections in 8 mM EDTA (pH 8.0) at 98°C for 20 min. IHC staining for SULF2 was carried out using a *recently described* antibody which recognizes the 75 kDa amino-terminal fragment of SULF2 [27] according to a published protocol with minor modifications [7]. Briefly, SULF2 staining was performed using 8G1 monoclonal antibody and visualized using EnVision+™ horseradish peroxidase labeled polymer (K4001, Dako, Carpinteria, CA) according to the manufacturer's instructions. Slides were treated with 3% hydrogen peroxide and 1% bovine serum albumin (BSA) for 10 min each. Tissue sections were then exposed for 1 h at room temperature to the SULF2 mAb 8G1 diluted 1 in 50 in 1% BSA. Slides were then incubated with the EnVision+™ polymer for 30 min followed by DAB chromagen (Dako, Carpinteria, CA) for 5 min. Slides were counterstained with Harris modified Hematoxylin (Fisher, Pittsburgh, PA), blued in 1% ammonium hydroxide, dehydrated, and mounted with Acrymount (StatLab, McKinney, TX). Consecutive sections with the primary antibody omitted were used as negative controls and sections of non-small cell lung tumors served as positive controls [7]. All intermediate washes were performed with Tris-buffered saline with 0.05% Tween 20^©^ (Fisher, Pittsburg, PA).

IHC staining for the detection of p16, a protein associated with HPV infection [44], was carried out using the CINtec^©^ histology kit (Roche, Basel, Switzerland). The kit was used as directed by the manufacturer and includes the p16 monoclonal antibody E6H4, IgG_2a_ subtype produced in mouse and antigen retrieval solution. Samples were fixed, sectioned, de-paraffinized, detected and counter stained using the same methods as used for SULF2 IHC. The same conditions for antigen retrieval and negative controls were also used.

SULF2 staining in tumor and adjacent normal tissues were scored by the study pathologist. SULF2 and p16 staining were scored using the following method. An intensity score between 0 (no staining) and 3 (intense staining) was given. A score of the proportion of stained cells of 0 (0% cells stained), 1 (< 10% cells stained), 2 (10–50% cells stained) or 3 (> 50% cell stained) was also given. The combined score is the sum of the intensity and proportion of stained cells scores. All samples were blinded and randomized by coding at collection. This information was only made available to the data analyst after completion of the study.

### Protein extraction from mouthwash samples

Extracts were prepared from mouthwash samples (50 ml) for ELISA analysis, beginning by centrifugation at 250 × g for 20 min at 4°C. Supernatants were dialyzed against PBS, concentrated using Amicon Ultra 30 kDa MWCO concentrators (EMD Millipore, Billerica, MA), precipitated by acetone and reconstituted in PBS buffer (500 μl). Cell pellets were resuspended in 1 M NaCl solution containing cOmplete™, EDTA-free protease inhibitor cocktail (Roche, Indianapolis, IN) and treated by 5 freeze-thaw cycles. The extracts were cleared by centrifugation at 15,000 × g for 10 min at 4°C. Protein concentration of samples was determined by Bradford assay.

### SULF2 ELISA analysis

The sandwich ELISA method has been described in detail previously [27]. A SULF2 standard curve was produced using media conditioned by the MCF7 breast cancer cell line. The absolute amount of SULF2 in the conditioned media was determined by quantitative immunoblotting as previously described [2]. Two SULF2 monoclonal antibodies (mAb) were used in the sandwich ELISA, 5C12 as the capture antibody and biotinylated 8G1 as the detection antibody [27]. Both antibodies recognize the amino-terminal fragment of SULF2. Wells were washed with PBS containing 0.1% Tween 20 between each step. All steps were conducted at room temperature unless stated otherwise.

To quantify SULF2, 96-well plates were coated with 0.5 μg of 5C12 in 100 μl PBS overnight at 4°C, and isotype specific control wells coated with mouse IgG1 (Affymetrix eBioscience, San Diego, CA). Blocking of non-specific sites with 3% BSA in PBS for 1 h followed. Serum (20 μl) or extracted mouthwash supernatant samples were prepared by diluting to 100 μl/well with 1% BSA in PBS and allowed to incubate for 30 min with 25 μg/ml polyclonal mouse IgG (Sigma-Aldrich, St. Louis, MO) to counteract anti-mouse antibodies that are present in some subjects. The prepared samples were transferred to coated plates for 1 h to capture SULF2. The detection antibody (100 μg biotinylated 8G1 (2 μg/ml) in 1% BSA in PBS containing 25 μg/ml polyclonal mouse IgG) was added for 1 h followed by 10 ng of streptavidin-HRP (Jackson ImmunoResearch, West Grove, PA) in 100 μl 1% BSA in PBS with 0.05% Tween 20, for 30 min. Plates were developed with TMB substrate before quenching with 0.2 M H_2_SO_4_. SULF2 levels in mouthwash extracts were normalized to total protein in the samples as determined by the Bradford assay.

### Western blot analysis

SULF2 detection in mouthwash supernatant and pellet was confirmed by Western blot analysis. Mouthwash samples were incubated with LDS sample buffer and 100 mM DTT for 10 min at 95°C before loading onto 4–12% Bis-Tris gels and transfer to PVDF membrane. Membranes were blocked with casein blocking buffer (Sigma-Aldrich, St. Louis, MO) for 1 hr and probed with 8G1 at a concentration of 4 μg/ml overnight at 4°C. All intermediary washes used PBS with 0.05% Tween-20. Rabbit anti-mouse IgG peroxidase conjugated (A9044, Sigma-Aldrich, St. Louis, MO) secondary antibody was diluted 1 in 100 000 in PBS-T and incubated for 1 hr followed by washing and visualization using Clarity™ Western ECL substrate (BIO-RAD, Hercules, CA).

### Statistical analyses

Data are expressed as means and standard deviations (SD) or frequencies. Differences in the intensity of SULF2 staining, the proportion of stained cells and the combined scores of HNSCC tissue between sections containing tumor and its adjacent cancer-free regions, were evaluated using independent samples *t*-test. Separate analyses were also conducted by stage among cases categorized as early stage (stage 1 and 2) and advanced stage (stage 3 and 4). Differences in mean SULF2 combined scores between categories defined by tumor location, age, gender, race, smoking, and HPV infection among both cancer-free controls and cancer cases were assessed using independent samples *t*-test or one-way ANOVA.

Analyses were also conducted for SULF2 concentration in serum and mouthwash among cancer-free controls and HNSCC patients. SULF2 concentrations were not normally distributed in serum and a small sample size of mouthwash was included in the study. Hence, Wilcoxon rank-sum test was used to evaluate average differences in serum and mouthwash separately. One high outlier value in serum was excluded from the healthy group based on the Grubbs test (*p* < 0.01). Correlations between age and serum concentrations were determined with the Spearman rank correlation for non-normally distributed values. All statistical tests were based on a two-sided *p* value. Tests with *p* values < 0.05 were considered statistically significant unless specified otherwise. SAS version 9.4 (SAS Inst. Inc., Cary. NC) and GraphPad Prism version 6 for windows (GraphPad Software, La Jolla, CA) were used for statistical analysis and preparation of graphs.

## SUPPLEMENTARY FIGURES AND TABLES


