# Supplementary material for: Expression of the extracellular sulfatase SULF2 is associated with squamous cell carcinoma of the head and neck
Source: Oncotarget. 2016 May 20;7(28):43177–87. doi: 10.18632/oncotarget.9506 (PMC5190016; doi:10.18632/oncotarget.9506)
Supplement: Supplementary file 1 [file oncotarget-07-43177-s001.pdf]

Expression of the extracellular sulfatase SULF2 is associated with squamous cell carcinoma of the head and neck

Supplementary Materials

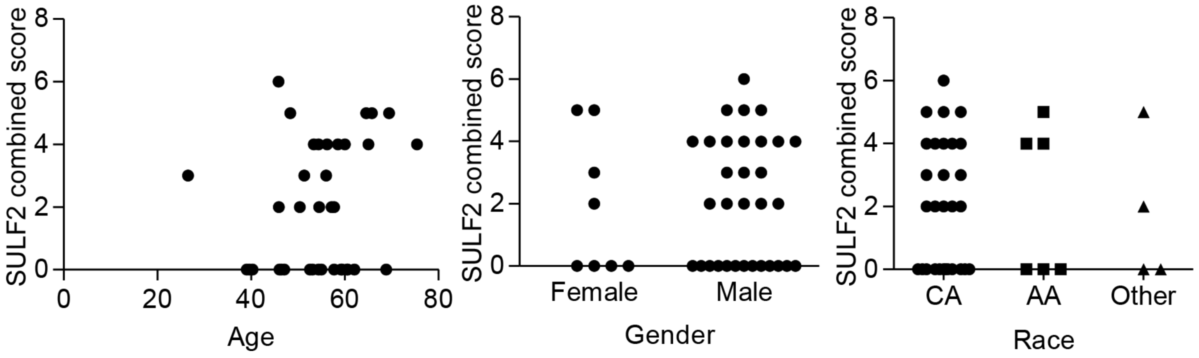

Supplementary Figure S1: Association between SULF2 staining and patient demographics (A) age (B) gender and (C) race. CA is Caucasian; AA is African American; remaining participants are of other racial categories or of unknown race. SULF2 staining is shown as the combined score of the intensity and the proportion of cells stained.

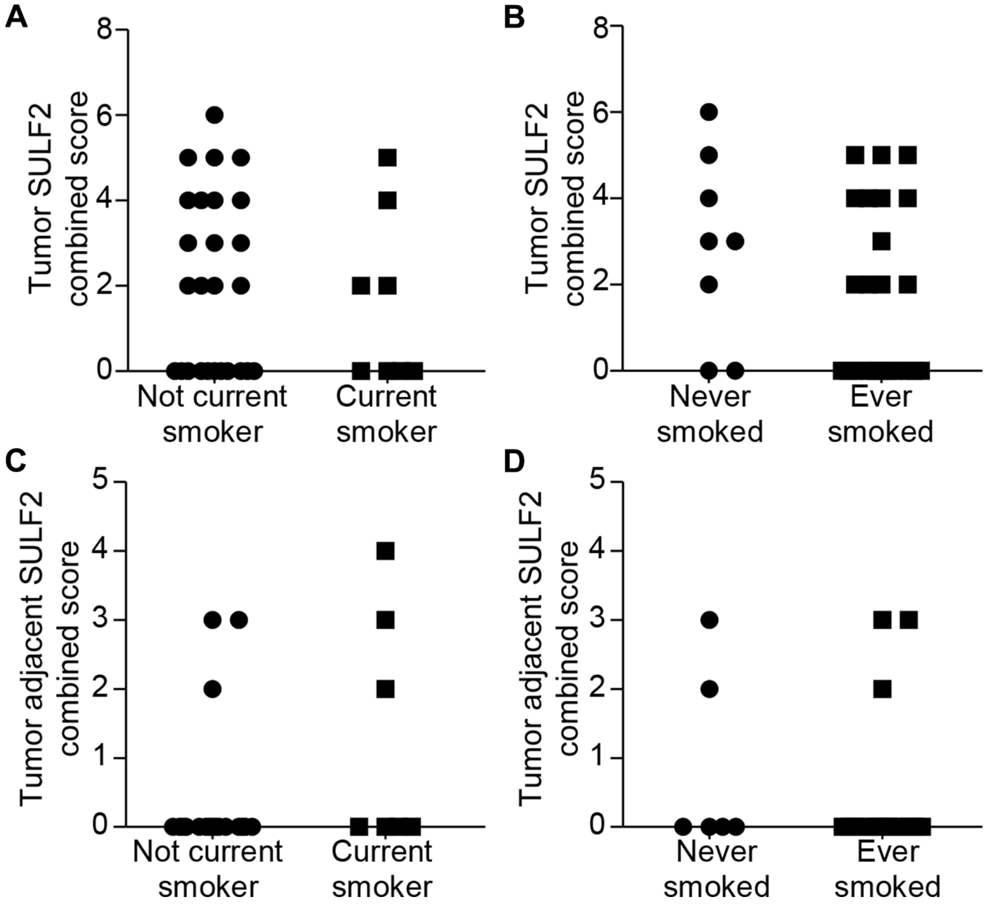

Supplementary Figure S2: Association between SULF2 staining in tumor tissue and smoking status. (**A**) Current smoker (n = 8) vs ex- and non-smoker (n = 28) (p-value = 0.49). (**B**) Ever smoker (n = 25) vs non-smoker (n = 8) (p-value = 0.21). Association between SULF2 staining and smoker status in tissue adjacent to tumor. (**C**) Current smoker (n = 18) vs ex- and non-smoker (n = 7) (p-value 0.31). (**D**) Ever smoker (n = 18) vs non-smoker (n = 6) (p-value 0.63). SULF2 staining is shown as the combined score of the intensity and the proportion of cells stained.

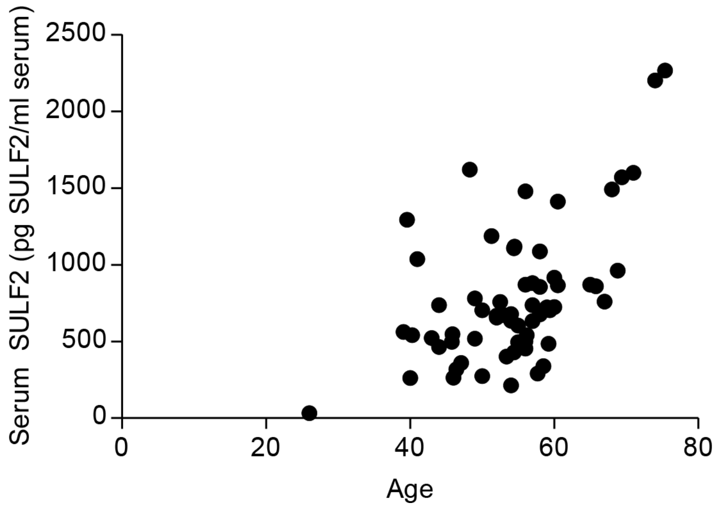

Supplementary Figure S3: Association between age and concentration of SULF2 in serum (Spearman r = 0.47 and p-value < 0.001). As no difference was observed between controls and HNSCC patients, all patients were grouped.
